# Intelectin 1 suppresses the growth, invasion and metastasis of neuroblastoma cells through up-regulation of N-myc downstream regulated gene 2

**DOI:** 10.1186/s12943-015-0320-6

**Published:** 2015-02-21

**Authors:** Dan Li, Hong Mei, Jiarui Pu, Xuan Xiang, Xiang Zhao, Hongxia Qu, Kai Huang, Liduan Zheng, Qiangsong Tong

**Affiliations:** Department of Pediatric Surgery, Union Hospital, Tongji Medical College, Huazhong University of Science and Technology, Wuhan, Hubei Province 430022 P. R. China; Clinical Center of Human Genomic Research, Union Hospital, Tongji Medical College, Huazhong University of Science and Technology, Wuhan, Hubei Province 430022 P. R. China; Department of Pathology, Union Hospital, Tongji Medical College, Huazhong University of Science and Technology, Wuhan, Hubei Province 430022 P. R. China

**Keywords:** Neuroblastoma, Intelectin 1, N-myc downstream regulated gene 2, Tumorigenesis, Aggressiveness

## Abstract

**Background:**

Recent studies have revealed the potential roles of intelectin 1 (ITLN1) in tumorigenesis. However, its functions and underlying mechanisms in neuroblastoma (NB), the most common extracranial solid tumor in childhood, still remain largely unknown.

**Methods:**

Human neuroblastoma cell lines were treated with recombinant ITLN1 protein or stably transfected with *ITLN1* expression and short hairpin RNA vectors. Gene expression and signaling pathway were detected by western blot and real-time quantitative RT-PCR. Gene promoter activity and transcription factor binding were detected by luciferase reporter and chromatin immunoprecipitation assays. Growth and aggressiveness of tumor cells were measured by MTT colorimetry, colony formation, scratch assay, matrigel invasion assay, and nude mice model.

**Results:**

Mining of public microarray databases revealed that N-myc downstream regulated gene 2 (NDRG2) was significantly correlated with ITLN1 in NB. Gain- and loss-of-function studies indicated that secretory ITLN1 facilitated the NDRG2 expression, resulting in down-regulation of vascular endothelial growth factor (VEGF) and matrix metalloproteinase 9 (MMP-9), in NB cell lines SH-SY5Y, SK-N-BE(2), and SK-N-SH. Krüppel-like factor 4 (KLF4), a transcription factor crucial for NDRG2 expression, was up-regulated by ITLN1 in NB cells via inactivation of phosphoinositide 3-kinase (PI3K)/AKT signaling. Ectopic expression of *ITLN1* suppressed the growth, invasion and metastasis of NB cells *in vitro* and *in vivo*. Conversely, knockdown of *ITLN1* promoted the growth, invasion, and metastasis of NB cells. In addition, rescue experiments in ITLN1 over-expressed or silenced NB cells showed that restoration of NDRG2 expression prevented the tumor cells from ITLN1-mediated changes in these biological features. In clinical NB tissues, ITLN1 was down-regulated and positively correlated with NDRG2 expression. Patients with high ITLN1 or NDRG2 expression had greater survival probability.

**Conclusions:**

These findings indicate that ITLN1 functions as a tumor suppressor that affects the growth, invasion and metastasis of NB through up-regulation of NDRG2.

**Electronic supplementary material:**

The online version of this article (doi:10.1186/s12943-015-0320-6) contains supplementary material, which is available to authorized users.

## Background

Neuroblastoma (NB), the most common extracranial solid tumor in childhood, accounts for 15% of all pediatric cancer deaths [1]. For patients with high-risk NB, despite the application of many therapeutic modalities, such as surgery, chemoradiotherapy, stem cell transplantation, and immunotherapy, the prognosis still remains dismal [1]. Recent evidence indicates that galectins, a family of animal lectins, are aberrantly expressed in tumor tissues and play crucial roles in neoplastic transformation and the growth, migration, invasion, and metastasis of tumor cells [2]. For example, inhibition of galectin-1 expression significantly suppresses the transformed phenotypes of human glioma cells [3]. Over-expression of galectin-3 into human T lymphoma Jurkat cells results in faster growth *in vitro* [2], while inhibition of galectin-3 expression attenuates the growth of breast carcinoma and thyroid papillary carcinoma cells [4,5]. Previous evidence indicates that both galectin-1 and galectin-7 inhibit the growth of NB cells [6,7], while galectin-3 is broadly expressed in NB cells to impair the apoptosis-sensitive phenotype induced by MYCN [8]. However, the roles of other lectins in the progression and aggressiveness of NB still remain largely unknown and warrant further investigation.

Intelectin 1 (ITLN1) is a novel identified secretory and galactose-binding lectin that is expressed in the heart, small intestine, colon, kidney collecting tubule cells, bladder umbrella cells, and some mesothelial cells [9,10]. It has been reported that ITLN1 participates in the immune defense against microorganisms [9], and is related to chronic obstructive pulmonary disease [11] and asthma [12]. ITLN1 also participates in insulin-stimulated glucose uptake in human subcutaneous and omental adipocytes [13]. More importantly, recent evidence shows the emerging roles of ITLN1 in tumorigenesis. ITLN1 is over-expressed in human malignant pleural mesothelioma (MPM) and secreted into pleural effusions, and serves as a biomarker for differentiating from lung cancer [14,15]. Our previous studies have shown that ITLN1 is aberrantly expressed in gastric cancer tissues, and is correlated with clinicopathological features, suggesting its value as a useful prognostic factor for gastric cancer patients [16]. However, the expression profiles, exact functions, and downstream targets of ITLN1 in NB still remain elusive. In the current study, we demonstrate, for the first time, that ITLN1 is down-regulated in NB tissues and cell lines. Secretory ITLN1 suppresses the growth, invasion, and metastasis of NB cells *in vitro* and *in vivo* through up-regulating N-myc downstream regulated gene 2 (NDRG2). In addition, the expression of Krüppel-like factor 4 (KLF4), a transcription factor responsible for the up-regulation of NDRG2, was enhanced by ITLN1 in NB cells, suggesting the crucial roles of ITLN1 in the progression and aggressiveness of NB.

## Results

### ITLN1 facilitates the NDRG2 expression at transcriptional levels in NB cells

Mining the publicly available clinical tumor expression datasets [R2: microarray analysis and visualization platform (http://hgserver1.amc.nl/cgi-bin/r2/main.cgi)] revealed the decreased *ITLN1* transcript levels in some kinds of cancer, including colon cancer, lung cancer, renal cancer, prostate cancer, and NB (Additional file 1: Figure S1A). Further analysis revealed six over-lapping genes significantly correlated with ITLN1 in these cancers (Additional file 1: Figure S1B), including *NDRG2*, chaperonin containing TCP1 subunit 3 (*CCT3*), defective in cullin neddylation 1 domain containing 5 (*DCUN1D5*), enolase 1 (*ENO1*), microtubule-actin crosslinking factor 1 (*MACF1*), and Mg^2+^/Mn^2+^ dependent protein phosphatase 1G (*PPM1G*). The *ITLN1* and *NDRG2* transcript levels in NB tissues were positively correlated (correlation coefficient *R* = 0.291, *P* = 0.0059, Additional file 1: Figure S1C), and were inversely associated with the international neuroblastoma staging system (INSS) stages (Additional file 1: Figure S1D).

**Figure 1:**
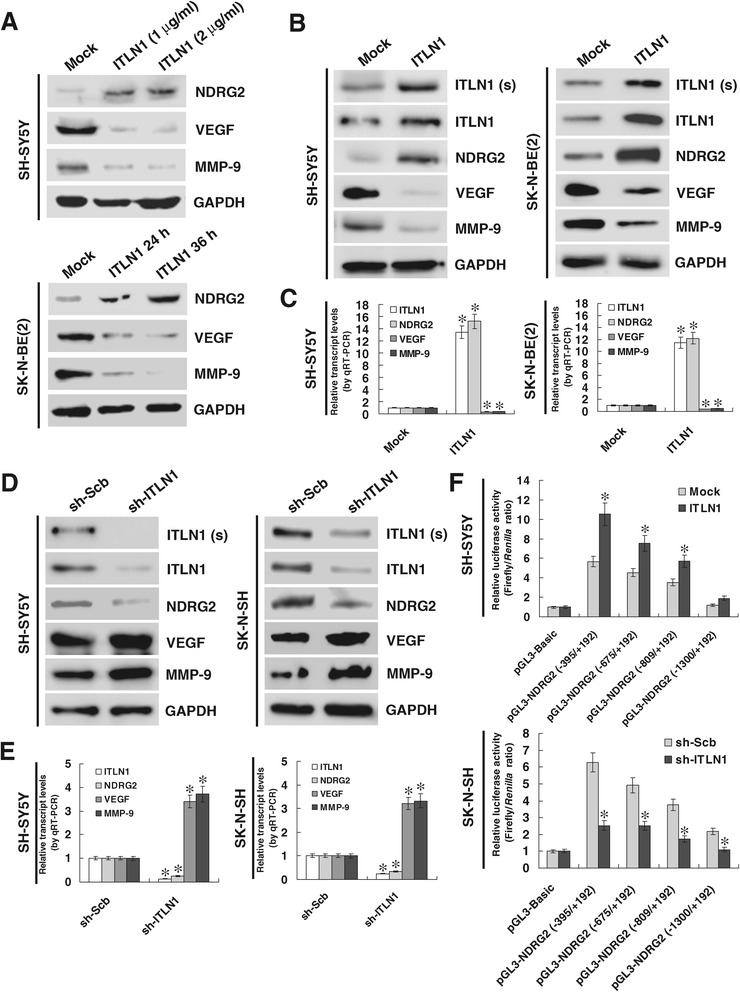
**ITLN1 facilitates the NDRG2 expression in NB cells. (A)** Western blot showing the expression of NDRG2, VEGF, and MMP-9 in solvent (mock)- or recombinant ITLN1-treated SH-SY5Y (1 and 2 μg/ml, for 24 hrs) and SK-N-BE(2) (1 μg/ml, for 24 and 36 hrs) cells. **(B)** Western blot showing the protein levels of ITLN1 [in culture supernatant (s) and lysate], NDRG2, VEGF, and MMP-9 (in lysate) in NB cells stably transfected with empty vector (mock) or *ITLN1*. **(C)** The transcript levels of *ITLN1*, *NDRG2*, *VEGF*, and *MMP-9* in NB cells stably transfected with mock or *ITLN1* as measured by real-time quantitative RT-PCR. **(D)** Western blot showing the expression of ITLN1 [in culture supernatant (s) and lysate], NDRG2, VEGF, and MMP-9 (in lysate) in SH-SY5Y and SK-N-SH cells stably transfected with sh-Scb or sh-ITLN1. **(E)** The transcript levels of *ITLN1*, *NDRG2*, *VEGF*, and *MMP-9* in NB cells stably transfected with sh-Scb or sh-ITLN1 as detected by real-time quantitative RT-PCR. **(F)** Luciferase reporter assay showing the activity of different *NDRG2* promoter fragments in NB cells stably transfected with mock, *ITLN1*, sh-Scb, or sh-ITLN1. *P < 0.01 vs. mock or sh-Scb.

To address the hypothesis that ITLN1 might influence the NDRG2 expression in NB, recombinant ITLN1 protein was administrated into cultured NB cell lines SH-SY5Y and SK-N-BE(2). As shown in Figure 1A, either low dose (1 μg/ml) or high dose (2 μg/ml) of recombinant ITLN1 protein markedly induced the expression of NDRG2 in NB cells at 24 and 36 hrs post-administration. In addition, *ITLN1* vector was stably transfected into SH-SY5Y and SK-N-BE(2) cells, resulting in enhanced ITLN1 expression and secretion into culture supernatant and increased NDRG2 expression levels, than those stably transfected with empty vector (mock) (Figure 1B and C). In addition, the expression of vascular endothelial growth factor (*VEGF*) and matrix metallopeptidase 9 (*MMP-9*), downstream target genes of NDRG2 [17,18], was also decreased in NB cells treated with recombinant ITLN1 protein or stably transfected with *ITLN1* (Figure 1A, B and C). Since over-expression of NDRG2 suppressed the expression of VEGF and MMP-9 in NB cells (Additional file 2: Figure S2A), and knockdown of NDRG2 rescued ITLN1-induced down-regulation of VEGF and MMP-9 (Additional file 2: Figure S2B), we believed that ITLN1 regulated the expression of VEGF and MMP-9 through modulating NDRG2. On the other hand, stable transfection of sh-ITLN1 into SH-SY5Y and SK-N-SH cells resulted in obviously reduced expression and secretion of ITLN1, decreased NDRG2 levels, and increased expression of VEGF and MMP-9 than those of scramble short hairpin RNA (sh-Scb)-transfected cells (Figure 1D and E). In addition, stable over-expression or knockdown of ITLN1 resulted in increased and decreased *NDRG2* promoter activity in NB cells, respectively, especially at −395/+192 bp region relative to the transcription start site (TSS) (Figure 1 F). In contrast, the expression of other potential target genes analyzed by R2: microarray analysis and visualization platform, including *CCT3*, *DCUN1D5*, *ENO1*, *MACF1*, and *PPM1G*, was not affected by ITLN1 in NB cells (Additional file 3: Figure S3). Overall, these results demonstrate that ITLN1 considerably facilitates the transcription of *NDRG2* in NB cells.

### Involvement of KLF4 in ITLN1-mediated up-regulation of NDRG2

**Figure 2:**
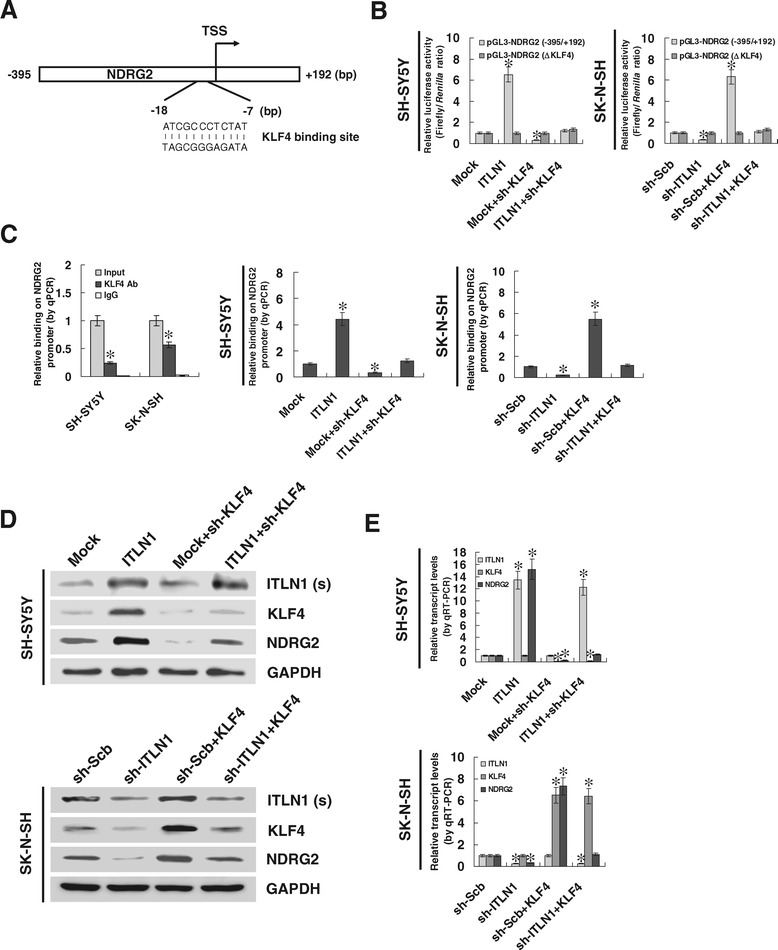
**Involvement of KLF4 in ITLN1-mediated up-regulation of NDRG2. (A)** Scheme of the potential binding site of KLF4 within *NDRG2* promoter. **(B)** Luciferase reporter assay showing the activity of *NDRG2* promoter and its mutant in NB cells stably transfected with empty vector (mock), *ITLN1*, sh-Scb, or sh-ITLN1, and those co-transfected with sh-KLF4 or *KLF4*. **(C)** ChIP and qPCR assays showing the binding of KLF4 on −133/+55 region of *NDRG2* promoter in NB cells stably transfected with mock, *ITLN1*, sh-Scb, or sh-ITLN1, and co-transfected with sh-KLF4 or *KLF4*. **(D)** Western blot showing the protein levels of ITLN1 [in culture supernatant (s)], KLF4, and NDRG2 (in lysate) in NB cells stably transfected with mock, *ITLN1*, sh-Scb, or sh-ITLN1, and those co-transfected with sh-KLF4 or *KLF4*. **(E)** The transcript levels of *ITLN1*, *KLF4*, and *NDRG2* in NB cells stably transfected with mock, *ITLN1*, sh-Scb, or sh-ITLN1, and those co-transfected with sh-KLF4 or *KLF4* as detected by real-time quantitative RT-PCR. *P < 0.01 vs. mock or sh-Scb.

To investigate the mechanisms underlying ITLN1-mediated up-regulation of NDRG2, we analyzed the transcription factor binding sites within *NDRG2* promoter, and noted one potential binding site of transcription factor KLF4, locating at bases 7–18 upstream the TSS (Figure 2A). The KLF4 levels were correlated with NDRG2 expression (correlation coefficient *R* = 0.441, *P* < 0.001) and greater survival probability (*P* = 0.027) in 88 NB cases derived from R2 microarray analysis and visualization platform (Additional file 4: Figure S4). Dual-luciferase assay indicated that ectopic expression or knockdown of KLF4 increased and decreased the promoter activity of *NDRG2* in NB cells, and the ITLN1-facilitated *NDRG2* promoter activity was abolished by mutation of KLF4 binding site (Figure 2B). In addition, chromatin immunoprecipitation (ChIP) and quantitative PCR (qPCR) indicated that over-expression or knockdown of ITLN1 increased or decreased the binding of KLF4 on −133/+55 region of *NDRG2* promoter in SH-SY5Y and SK-N-SH cells, which was rescued by knockdown or ectopic expression of KLF4, respectively (Figure 2C). Moreover, western blot and real-time quantitative RT-PCR indicated that ITLN1 up-regulated the protein levels, but not the transcript levels, of KLF4 in NB cells (Figure 2D and E), indicating that ITLN1 may facilitate the expression of KLF4 at the translational level. Knockdown or ectopic expression of KLF4 into NB cells prevented the NB cells from ITLN1-mediated changes in NDRG2 expression (Figure 2D and E). These results indicate that KLF4 facilitates the transcription of *NDRG2*, and plays a crucial role in ITLN1-induced up-regulation of NDRG2 in NB cells.

### ITLN1 facilitates the expression of KLF4 via inactivation of PI3K/AKT signaling

**Figure 3:**
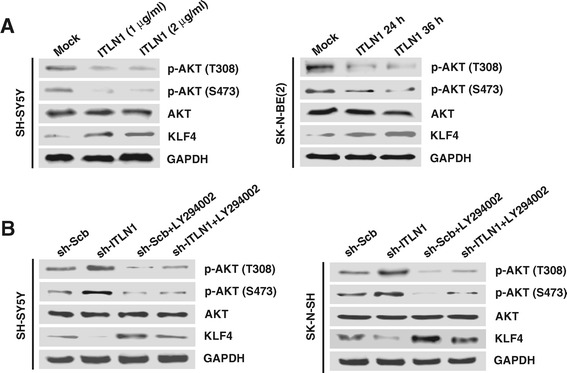
**ITLN1 facilitates the expression of KLF4 via inactivation of PI3K/AKT signaling. (A)** Western blot showing the phosphorylation of AKT (T308 and S473) and expression of KLF4 in solvent (mock)- or recombinant ITLN1-treated SH-SY5Y (1 and 2 μg/ml for 24 hrs) and SK-N-BE(2) (1 μg/ml for 24 and 36 hrs) cells. **(B)** Western blot showing the phosphorylation of AKT (T308 and S473) and expression of KLF4 in NB cells stably transfected with sh-Scb or sh-ITLN1, and those pre-treated with LY294002 (10 μmol/L).

To further explore the mechanisms for ITLN1-induced KLF4 expression, we observed the changes in phosphoinositide 3-kinase (PI3K)/AKT signaling that regulates the KLF4 expression [19]. Administration of recombinant ITLN1 protein (1 and 2 μg/ml) into SH-SY5Y and SK-N-BE(2) cells reduced the phosphorylation of AKT (T308 and S473), and up-regulated the expression of KLF4 at 24 and 36 hrs post-administration, than those treated with solvent control (mock) (Figure 3A). In contrast, stable knockdown of ITLN1 induced the phosphorylation of AKT (T308 and S473), and down-regulated the KLF4 expression in SH-SY5Y and SK-N-SH cells, which was abolished by administration of PI3K activity inhibitor LY294002 (Figure 3B). These results suggest that ITLN1 facilitates the KLF4 expression through attenuating PI3K/AKT signaling in NB cells.

### Ectopic expression of ITLN1 suppresses the growth, migration and invasion of NB cells through up-regulating NDRG2

**Figure 4:**
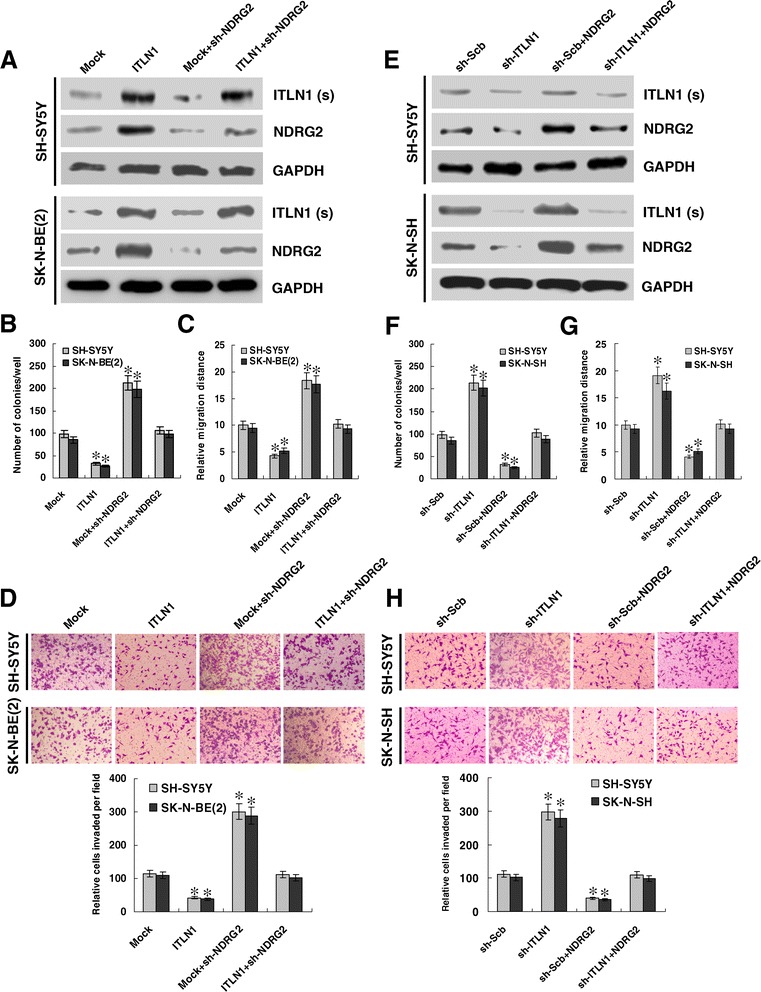
**ITLN1 suppresses the growth, migration, and invasion of NB cells** ***in vitro*** **through up-regulating NDRG2. (A and E)** Western blot showing the protein levels of ITLN1 [in culture supernatant (s)] and NDRG2 (in lysate) in NB cells stably transfected with empty vector (mock), *ITLN1*, sh-Scb, or sh-ITLN1, and those co-transfected with sh-NDRG2 or *NDRG2*. **(B and F)** Quantification of colony formation assay showing the growth potential of NB cells stably transfected with mock, *ITLN1*, sh-Scb, or sh-ITLN1, and those co-transfected with sh-NDRG2 or *NDRG2*. **(Cand G)** Migration of NB cells upon transfection with mock, *ITLN1*, *NDRG2*, sh-Scb, sh-ITLN1, or sh-NDRG2 depicted by scratch assay after 24 hrs. **(D and H)** Representation (top) and quantification (bottom) of matrigel invasion assay showing the *in vitro* invasion of NB cells stably transfected with mock, *ITLN1*, sh-Scb, or sh-ITLN1, and those co-transfected with sh-NDRG2 or *NDRG2*. *P < 0.01 vs. mock or sh-Scb.

We further investigated the effects of ITLN1 over-expression and target gene restoration on cultured NB cells. Transfection of short hairpin RNA (shRNA) targeting *NDRG2* restored the ITLN1-induced up-regulation of NDRG2 in SH-SY5Y and SK-N-BE(2) cells (Figure 4A and Additional file 5: Figure S5A). In line with the results from MTT colorimetric assay (Additional file 6: Figure S6A), colony formation assay indicated that ITLN1 over-expression attenuated the growth of SH-SY5Y and SK-N-BE(2) cells, when compared to those stably transfected with empty vector (mock) (Figure 4B and Additional file 6: Figure S6B). In scratch assay, ITLN1 over-expression attenuated the migration capabilities of SH-SY5Y and SK-N-BE(2) cells (Figure 4C and Additional file 6: Figure S6C). Transwell analysis showed that NB cells stably transfected with ITLN1 presented an impaired invasion capacity than mock cells (Figure 4D). In addition, restoration of NDRG2 expression rescued the NB cells from their changes in these phenotypes induced by stable over-expression of ITLN1 (Figure 4B, C, and D, Additional file 6: Figure S6A, B, and C). These results reveal the tumor suppressive roles of ITLN1, and indicate that up-regulation of NDRG2 is involved in the ITLN1-inhibited aggressiveness of NB cells.

### Knockdown of ITLN1 promotes the growth, migration, and invasion of NB cells *in vitro*

To further explore the influence of ITLN1 on the aggressiveness of NB cells, we investigated the effects of ITLN1 knockdown and NDRG2 restoration on cultured NB cells. Transfection of *NDRG2* restored the down-regulation of NDRG2 induced by ITLN1 knockdown in SH-SY5Y and SK-N-SH cells (Figure 4E and Additional file 5: Figure S5B). In MTT colorimetric and colony formation assays, knockdown of ITLN1 facilitated the viability and growth of SH-SY5Y and SK-N-SH cells, than those stably transfected with sh-Scb (Figure 4F, Additional file 6: Figure S6D and E). In scratch assay, ITLN1 knockdown increased the migration capabilities of SH-SY5Y and SK-N-SH cells (Figure 4G and Additional file 6: Figure S6F). Transwell analysis showed that NB cells stably transfected with sh-ITLN1 presented an increased invasion capacity (Figure 4H). In addition, restoration of NDRG2 expression rescued the SH-SY5Y and SK-N-SH cells from their changes in these phenotypes induced by stable knockdown of ITLN1 (Figure 4F, G, and H, Additional file 6: Figure S6D, E, and F). These findings further indicate the tumor suppressive roles of ITLN1 in regulating the growth, migration, and invasion of NB cells.

### ITLN1 suppresses the growth and metastasis of NB cells *in vivo*

**Figure 5:**
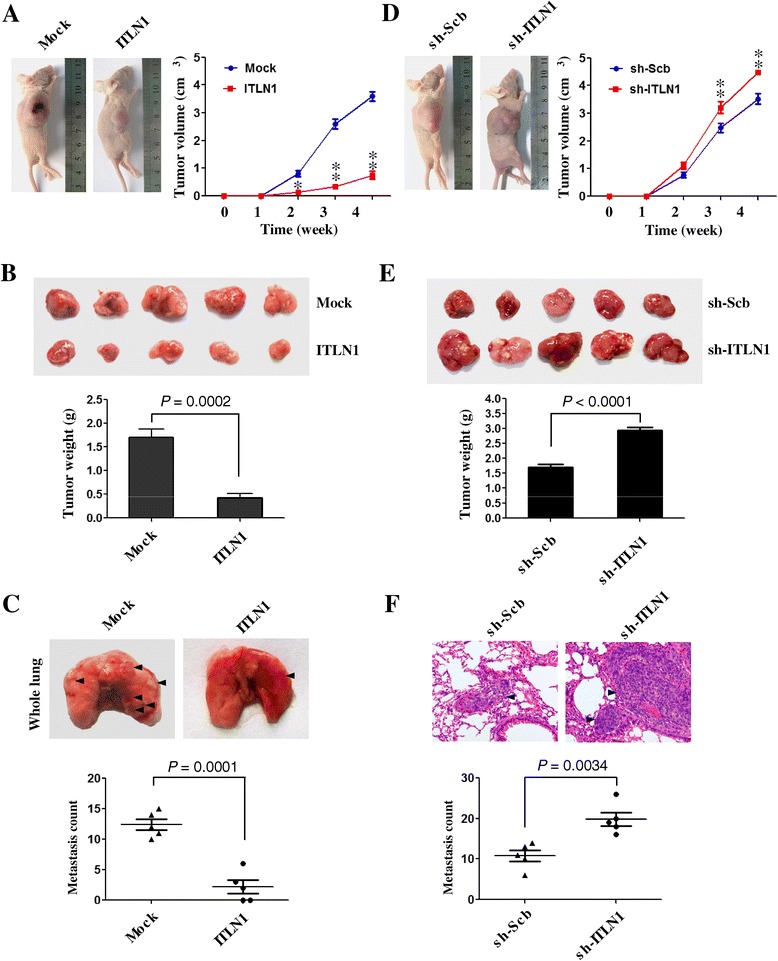
**ITLN1 attenuates the growth and metastasis of NB cells** ***in vivo*** **. (A and D)** Tumor growth curve of SH-SY5Y cells (1 × 10^6^) stably transfected with empty vector (mock), *ITLN1*, sh-Scb, or sh-ITLN1 in athymic nude mice (n = 5 for each group), after hypodermic injection for 4 weeks. **(B and E)** Representation (top) and quantification (bottom) of xenograft tumors formed by hypodermic injection of SH-SY5Y cells stably transfected with mock, *ITLN1*, sh-Scb, or sh-ITLN1. **(C and F)** Representation (top, arrowhead) and quantification (bottom) of lung metastasis after injection of SH-SY5Y cells (0.4 × 10^6^) stably transfected with mock, *ITLN1*, sh-Scb, or sh-ITLN1 into the tail vein of athymic nude mice (n = 5 for each group). **P < 0.001 vs. mock or sh-Scb.

We next investigated the efficacy of ITLN1 against tumor growth and metastasis *in vivo*. Stable transfection of ITLN1 into SH-SY5Y cells resulted in decreased growth and tumor weight of subcutaneous xenograft tumors in athymic nude mice, when compared to those stably transfected with empty vector (mock) (Figure 5A and B). In the experimental metastasis studies, SH-SY5Y cells stably transfected with *ITLN1* established statistically fewer lung metastatic colonies than mock group (Figure 5C). On the other hand, stable knockdown of ITLN1 in SH-SY5Y cells resulted in increased growth and tumor weight of subcutaneous xenograft tumors in athymic nude mice (Figure 5D and E), and more lung metastatic colonies (Figure 5F), than those stably transfected with sh-Scb. These results are consistent with the findings that ITLN1 suppresses the growth, migration, and invasion of NB cells *in vitro.* Accordingly, identification of *NDRG2* as the target gene of ITLN1 may explain, at least in part, why over-expression of ITLN1 suppresses the aggressiveness of NB.

### ITLN1 is under-expressed and inversely correlated with NDRG2 in NB tissues and cell lines

**Figure 6:**
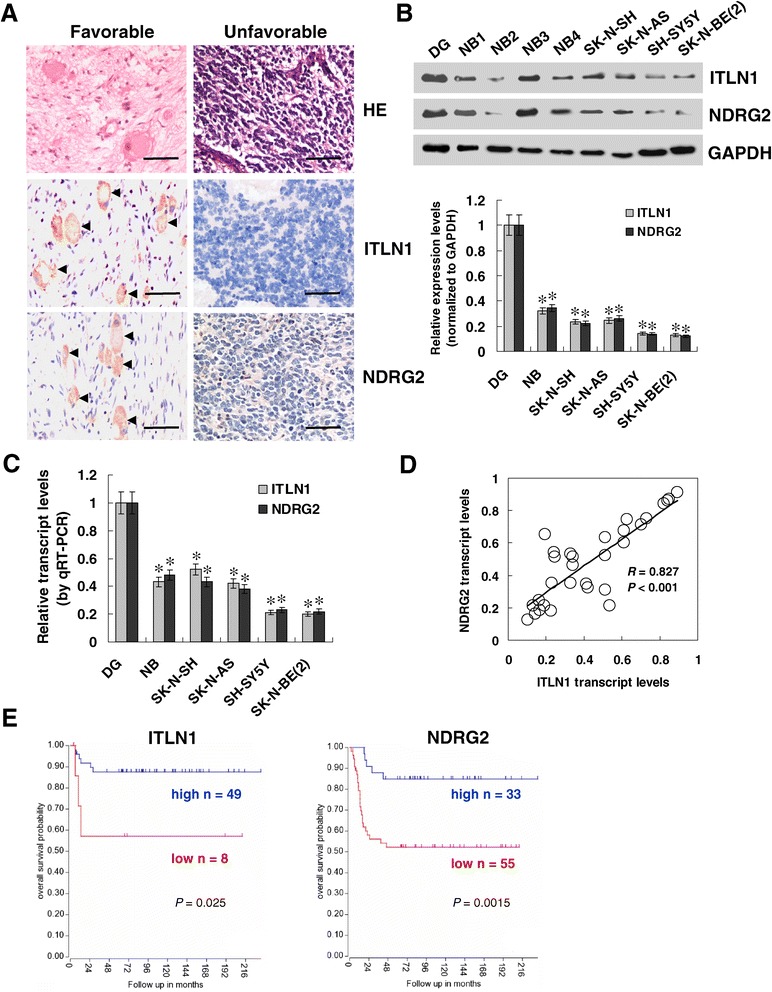
**ITLN1 is under-expressed in NB tissues and cell lines. (A)** Immunohistochemical staining showing the expression of ITLN1 and NDRG2 in tumor specimens from 42 NB cases (arrowheads, brown). Scale bars: 100 μm. **(B)** Western blot showing the endogenous protein levels of ITLN1 and NDRG2 in lysate from NB tissues (n = 30), NB cell lines, and normal dorsal ganglia (DG). **(C)** Endogenous transcript levels of *ITLN1* and *NDRG2* in NB tissues (n = 30), NB cell lines, and DG as detected by real-time quantitative RT-PCR. **(D)** Pearson’s coefficient correlation analysis for the relationship between *ITLN1* and *NDRG2* transcript levels in NB tissues (n = 30). **(E)** Kaplan–Meier survival plots of well-defined NB patients with low and high expression of ITLN1 or NDRG2, as derived from R2 microarray analysis and visualization platform (http://hgserver1.amc.nl/cgi-bin/r2/main.cgi). *P < 0.01 vs. DG.

To investigate the ITLN1 and NDRG2 expression in NB, paraffin-embedded sections from 42 well-established primary cases were collected [20,21]. Immunohistochemical staining revealed that ITLN1 was expressed in the tumor cells of NB tissues (Figure 6A). ITLN1 expression was detected in 14/42 (33.3%) cases and the staining was weak in 6, moderate in 5, and intense in 3 (Additional file 7: Table S1). The ITLN1 immunoreactivity was significantly higher in NB cases with age less than 1 year (*P* = 0.03), good differentiation (*P* < 0.001), lower mitosis karyorrhexis index (MKI) (*P* = 0.002), and early INSS stages (*P* = 0.003) (Additional file 7: Table S1). Notably, the immunostaining of ITLN1 was associated with NDRG2 immunoreactivity in NB cases (correlation coefficient *R* = 0.676, *P* < 0.001; Figure 6A and Additional file 8: Table S2). Moreover, western blot and real-time quantitative RT-PCR indicated lower expression levels of ITLN1 and NDRG2 in subtotal 30 NB specimens and cultured SK-N-SH, SK-N-AS, SH-SY5Y, and SK-N-BE(2) cell lines, than those in normal dorsal ganglia (Figure 6B and C). There was a positive correlation between *ITLN1* and *NDRG2* transcript levels in NB tissues (correlation coefficient *R* = 0.827, *P* < 0.001, Figure 6D). Administration of DNA methyltransferase inhibitor 5-aza-2′-deoxycytidine (5-Aza-CdR) or pan histone deacetylase inhibitor trichostatin A (TSA) resulted in slightly increased *ITLN1* transcript levels in NB cells (Additional file 9: Figure S7). Kaplan–Meier survival plots of well-defined NB cases derived from R2 microarray analysis and visualization platform revealed that patients with high ITLN1 (*P* = 0.025) or NDRG2 (*P* = 0.0015) expression had greater survival probability than those with low expression (Figure 6E). These results indicate that ITLN1 is under-expressed and correlated with the NDRG2 expression in NB tissues and cell lines.

## Discussion

Human *ITLN1* gene, locating at the chromosome 1q21.3 and encoding a glycoprotein consisting of 295 amino acids and N-terminal signal peptide (18 amino acids), was first isolated as the homolog of *Xenopus* oocyte lectin XL35 from a small intestine cDNA library [22]. Previous studies have shown that ITLN1 is a soluble protein detected in the culture supernatant of ITLN1-transfected cells [9]. Interestingly, over-expression of ITLN1 is identified in malignant pleural MPM by serial analysis of gene expression [15]. Epithelioid-type MPMs, but neither pleura-invading lung adenocarcinomas nor reactive mesothelial cells near the lung adenocarcinomas, are positive for ITLN1 immunostaining, suggesting that ITLN1 is a proper diagnostic marker for MPM [14]. Quantitative proteomic techniques have also revealed the value of ITLN1 as a useful proteomic tool for risk stratification and prediction of poor outcome in colorectal cancer [23]. Ectopic expression of ITLN1 into prostate cancer cells results in significantly decreased *in vitro* cell viability; meanwhile, increased tumorigenicity and *in vivo* growth are observed in ITLN1 knockdown prostate cancer cells, indicating a tumor suppressive role of ITLN1 in prostate cancer [24]. Recent evidence shows that ITLN1 significantly inhibits the proliferation and induces the apoptosis of hepatocellular carcinoma cells, via decreasing p53 deacetylation in a sirtuin 1-dependent manner [25]. These findings imply the potential roles of ITLN1 in the development and progression of human cancers. In the current study, we demonstrated the down-regulation of ITLN1 in clinical NB specimens, which was significantly associated with clinicopathological features and patients’ survival. Since administration of 5-Aza-CdR or TSA did not result in a significant increase in *ITLN1* transcript levels, we believe that the epigenetic mechanisms are not likely to be involved in the regulation of ITLN1 in NB cells. We further confirmed that secretory ITLN1 inhibited the growth, invasion, and metastasis of NB cells *in vitro* and *in vivo*, suggesting the tumor suppressive roles of ITLN1 in NB.

NDRG2, a member of the N-Myc downstream-regulated gene family, is down-regulated in many human cancers, such as breast cancer [26], liver cancer [27], and colorectal cancer [28], and exerts tumor suppressive functions associated with cell growth, invasion and metastasis [29]. Ectopic expression of NDRG2 inhibits the tumor growth through inducing suppressor of cytokine signaling 1 and subsequent inactivation of signal transducer and activator of transcription 3 in breast cancer cells [30] or by attenuating the AP-1 activity in colon carcinoma cells [31]. NDRG2 also inhibits the metastatic potentials of breast cancer cells through inducing bone morphogenetic protein 4 and subsequent suppression of MMP-9 expression [17]. NDRG2 modulates the adhesion and invasion of hepatocellular carcinoma cells through regulating CD24 expression [32]. In addition, NDRG2 suppresses the proliferation of breast cancer cells by reducing VEGF expression [18]. In this study, we demonstrated that NDRG2 was under-expressed in NB specimens and associated with patients’ survival, and NDRG2 suppressed the growth and aggressiveness of cultured NB cells. Moreover, restoration of NDRG2 expression prevented the NB cells from ITLN1-mediated changes in the growth, invasion, and metastasis, suggesting that ITLN1 may exert its tumor suppressive functions, at least in part, through up-regulating NDRG2 in NB.

KLF4 is a transcription factor that belongs to the Krüppel family of zinc finger proteins, and exhibits both oncogenic or tumor suppressive functions by interacting with the binding elements on promoters of target genes in different cellular contexts [33]. Tumor suppressive functions of KLF4 have been established in several human cancers, including colon cancer, gastric cancer, and bladder cancer [33]. KLF4 suppresses cell proliferation and promotes apoptosis through inducing cell cycle arrest at G1/S phase [34] and promoting p53-dependent activation of p21^Cip1^ [35]. Meanwhile, oncogenic properties of KLF4 have been indicated as its ability to reprogram fibroblast into pluripotent stem cells in cooperation with POU class 5 homeobox 1, sex-determining region Y-box 2, and c-Myc [36]. Previous studies indicate that KLF4 is under-expressed in NB tissues, and contributes to favorable disease outcome by directly mediating the growth and lineage determination of NB cells [37]. It has been established that PI3K/AKT signaling is required for the ubiquitination and degradation of KLF4 [19], and inhibition of AKT activation by PI3K inhibitor LY294002 stimulates the KLF4 expression through reducing its ubiquitination [19]. Our data showed that transcription factor KLF4 was crucial for the NDRG2 expression in NB cells. In addition, we found that ITLN1 induced the KLF4 expression via inactivation of PI3K/AKT signaling, which was required for ITLN1-mediated up-regulation of NDRG2 in NB cells, suggesting the tumor suppressive roles of ITLN1/KLF4/NDRG2 axis in the tumorigenesis of NB. Interestingly, we noted the physical interaction between ITLN1 and glucose-regulated protein 78 (GRP78) in public database BioGRID (http://thebiogrid.org/). GRP78 is an endoplasmic reticulum lumenal protein that localizes to the cell surface in cancer cells, and serves as a co-receptor for growth and survival signaling [38,39]. Since cell surface GRP78 forms complex with PI3K to promote the production of phosphatidyl inositol-3,4,5-triphosphate (PIP3) and subsequent PI3K/AKT signaling [40], we suspect that ITLN1 may modulate the PI3K/AKT signaling through interacting with GRP78 and regulating its activity in NB cells, which warrants our further investigation.

## Conclusions

In summary, for the first time, we have demonstrated that ITLN1 is down-regulated in human NB, and secretory ITLN1 efficiently inhibits the growth, invasion, and metastasis of NB cells *in vitro* and *in vivo* through up-regulating the expression of NDRG2. This study extends our knowledge about the regulation of tumor suppressive genes associated with the progression of NB, and suggests that ITLN1 may be of potential values as a novel therapeutic target for NB.

## Methods

### Patient tissue samples

Approval to conduct this study was obtained from the Institutional Review Board of Tongji Medical College (approval number: 2011-S085). Paraffin-embedded specimens from 42 well-established primary NB cases were obtained from the Department of Pediatric Surgery, Union Hospital of Tongji Medical College [20,21]. The pathological diagnosis of NB was confirmed by at least two pathologists. Based on the Shimada classification system, including MKI, degree of neuroblastic differentiation and stromal maturation, and patient’s age, 19 patients were classified as having favorable histology and 23 as having unfavorable histology. According to the INSS, 7 patients were classified as stage 1, 7 as stage 2, 9 as stage 3, 11 as stage 4, and 8 as stage 4S. Fresh tumor specimens were collected at surgery and stored at −80°C until use. Protein and RNAs of normal human dorsal ganglia were obtained from Clontech (Mountain View, CA).

### Immunohistochemistry

Immunohistochemical staining was performed as previously described [20,21], with antibodies specific for ITLN1 (Abcam, Cambridge, MA; Santa Cruz Biotechnology, Santa Cruz, CA; 1:200 dilutions) and NDRG2 (Santa Cruz Biotechnology; 1:200 dilution). The negative controls included parallel sections treated with omission of the primary antibody, in addition to an adjacent section of the same block in which the primary antibody was replaced by rabbit polyclonal IgG (Abcam Inc.) as an isotype control. The immunoreactivity in each tissue section was assessed by at least two pathologists without knowledge of the clinicopathological features of tumors. The degree of positivity was initially classified according to the percentage of positive tumor cells as the following: (−) < 5% cells positive, (1+) 6–25% cells positive, (2+) 26–50% cells positive, and (3+) >50% cells positive.

### Western blot

Tissue or cellular protein was extracted with 1× cell lysis buffer (Promega, Madison, WI). Culture supernatant was concentrated using a 10,000 MWCO spin column (Millipore, Billerica, MA). Protein expression in lysate or supernatant was analyzed by western blot as previously described [20,21,41-44], with antibodies specific for ITLN1, NDRG2, VEGF, MMP-9, p-AKT (T308), p-AKT (S473), AKT, KLF4, and glyceraldehyde-3-phosphate dehydrogenase (GAPDH, Santa Cruz Biotechnology). Enhanced chemiluminescence substrate kit (Amersham, Piscataway, NJ) was used for the chemiluminscent detection of signals with autoradiography film (Amersham).

### Real-time quantitative RT-PCR

Total RNA was isolated with RNeasy Mini Kit (Qiagen Inc., Valencia, CA). The reverse transcription reactions were conducted with Transcriptor First Strand cDNA Synthesis Kit (Roche, Indianapolis, IN). Real-time PCR was performed with SYBR Green PCR Master Mix (Applied Biosystems, Foster City, CA) and primers listed in Additional file 10: Table S3. The fluorescent signals were collected during extension phase, Ct values of the sample were calculated, and the transcript levels were analyzed by 2^-△△Ct^ method.

### Cell culture

Human NB cell lines SK-N-SH (HTB-11), SK-N-AS (CRL-2137), SH-SY5Y (CRL-2266), and SK-N-BE(2) (CRL-2271) were purchased from American Type Culture Collection (Rockville, MD). Cell lines were authenticated on the basis of viability, recovery, growth, morphology, and isoenzymology by the provider. Cell lines were used within 6 months after resuscitation of frozen aliquots, and grown in RPMI1640 medium (Life Technologies, Inc., Gaithersburg, MD) supplemented with 10% fetal bovine serum (Life Technologies, Inc.), penicillin (100 U/ml), and streptomycin (100 μg/ml). Cells were incubated in serum-free RPMI1640 for 4 hrs, and treated with recombinant ITLN1 protein (Enzo Life Sciences, Farmingdale, NY), LY294002 (Calbiochem, La Jolla, CA), 5-Aza-CdR (Sigma, St. Louis, MO), or TSA (Sigma) as indicated.

### Gene over-expression or knockdown

Human *ITLN1* cDNA (942 bp) and *KLF4* cDNA (1440) were amplified from NB tissue (Additional file 11: Table S4), and subcloned into pcDNA3.1 (Invitrogen, Carlsbad, CA). The oligonucleotides encoding shRNA specific for *ITLN1*, *NDRG2*, and *KLF4* (Additional file 11: Table S4) were subcloned into GV102 (Genechem Co., Ltd, Shanghai, China). Stable cell lines were screened by administration of neomycin (Invitrogen). The pcDNA3.1 and sh-Scb were applied as controls (Additional file 11: Table S4).

### Luciferase reporter assay

The *NDRG2* promoter luciferase reporter constructs were kindly provided by Dr. Jian Zhang [45]. Tumor cells were plated at 1 × 10^5^ cells/well on 24-well plates, and co-transfected with luciferase reporter vectors (30 ng) and *Renilla* luciferase reporter vector pRL-SV40 (10 ng, Promega). Twenty-four hrs post-transfection, firefly and *Renilla* luciferase activity were consecutively measured, according to the dual-luciferase assay manual (Promega). For *NDRG2* promoter activity, the luciferase signal was normalized by firefly/*Renilla* ratio.

### Rescue of target gene expression

Human *NDRG2* expression vector was provided by Dr. Victoria C. Foletta [46]. To restore the ITLN1-induced up-regulation of NDRG2, stable cell lines were transfected with the shRNA targeting the encoding region of *NDRG2* (Additional file 11: Table S4) by Genesilencer Transfection Reagent (Genlantis, San Diego, CA). The *NDRG2* expression vector was transfected into tumor cells stably transfected with shRNA specific for *ITLN1* (sh-ITLN1). The empty vector and sh-Scb were applied as controls, respectively (Additional file 11: Table S4).

### Chromatin immunoprecipitation

ChIP assay was performed according to the manufacturer’s instructions of EZ-ChIP kit (Upstate Biotechnology, Temacula, CA) [41,44,47]. DNA was sonicated into fragments of an average size of 200 bp. PCR primers were designed targeting the binding site of KLF4 within *NDRG2* promoter (Additional file 10: Table S3). Real-time qPCR with SYBR Green PCR Master Mix was performed using ABI Prism 7700 Sequence Detector. The amount of immunoprecipitated DNA was calculated in reference to a standard curve and normalized to input DNA.

### Cell viability assay

Tumor cells were cultured in 96-well plates at 5 × 10^3^ cells per well. Cell viability was monitored by the 2-(4,5-dimethyltriazol-2-yl)-2,5-diphenyl tetrazolium bromide (MTT, Sigma) colorimetric assay [41,47]. All experiments were done with 6–8 wells per experiment and repeated at least three times.

### Colony formation assay

Tumor cells were seeded at a density of 300 cells/ml on 35-mm dishes. Colony formation assay was performed as previously described [43,47,48]. Positive colony formation (more than 50 cells/colony) was counted. The survival fraction of cells was expressed as the ratio of plating efficiency of treated cells to that of control cells.

### Scratch migration assay

To minimize cell proliferation, tumor cells were starved in 0.5% serum medium, cultured in 24-well plates, and scraped with the fine end of 1-ml pipette tips (time 0). Plates were washed twice with phosphate buffered saline to remove detached cells, and incubated with the complete growth medium. Cell migration was photographed using 10 high-power fields, at 0, 24 hr post-induction of injury. Remodeling was measured as diminishing distance across the induced injury, normalized to the 0 hr control, and expressed as outgrowth (μm) [21,41,42,49].

### Cell invasion assay

Matrigel invasion assay was performed using membranes coated with Matrigel matrix (BD Science, Sparks, MD). To minimize the impacts of cell proliferation, homogeneous single cell suspensions (1 × 10^5^ cells/well) were starved in serum-free medium, added to the upper chambers, and allowed to invade for 24 hrs at 37°C in a CO_2_ incubator. Invaded cells were stained with 0.1% crystal violet for 10 min at room temperature and examined by light microscopy. Quantification of invaded cells was performed according to published criteria [20,21,41-43,50].

### *In vivo* growth and metastasis assay

All animal experiments followed the national guidelines for the care and use of animals, and were approved by the Animal Care Committee of Tongji Medical College (approval number: Y20080290). For the *in vivo* tumor growth studies, 2-month-old male nude mice (n = 5 per group) were injected subcutaneously in the upper back with 1 × 10^6^ tumor cells. One month later, mice were sacrificed and examined for tumor weight*.* The experimental metastasis (0.4 × 10^6^ tumor cells per mouse, n = 5 per group) studies were performed with 2-month-old male nude mice as previously described [20,21,44].

### Statistical analysis

Unless otherwise stated, all data were shown as mean ± standard error of the mean (SEM). The SPSS 18.0 statistical software (SPSS Inc., Chicago, IL) was applied for statistical analysis. The χ^2^ analysis and Fisher exact probability analysis were applied for comparison among the expression of ITLN1, NDRG2, and individual clinicopathological features. Pearson’s coefficient correlation was applied for analyzing the relationship between ITLN1 and NDRG2 expression. The Kaplan-Meier method was used to estimate survival rates, and the log-rank test was used to assess survival difference. Difference of tumor cells was determined by *t* test or analysis of variance (ANOVA).

## Additional files

Additional file 1: Figure S1. Data mining in R2: microarray analysis and visualization platform. **(A)** The *ITLN1* transcript levels in different types of normal and tumor tissues. **(B)** Over-lapping analysis showing six genes significantly correlated with ITLN1 in colon cancer, lung cancer, renal cancer, prostate cancer, and NB, including *NDRG2*, *CCT3*, *DCUN1D5*, *ENO1*, *MACF1*, and *PPM1G*. **(C)** Pearson’s coefficient correlation analysis for the relationship between *ITLN1* and *NDRG2* transcript levels in 88 well-defined NB cases. **(D)** The *ITLN1* and *NDRG2* transcript levels in NB cases (n = 88) with different INSS stages. **P* < 0.05 vs. normal tissues. Additional file 2: Figure S2. Direct regulation of VEGF and MMP-9 by NDRG2 in NB cells. **(A)** Western blot showing the expression of NDRG2, VEGF, and MMP-9 in SH-SY5Y and SK-N-BE(2) cells transfected with empty vector (mock) or *NDRG2*. **(B)** Western blot showing the expression of VEGF and MMP-9 in NB cells stably transfected with mock or *ITLN1*, and those co-transfected with sh-NDRG2. Additional file 3: Figure S3. ITLN1 does not affect the expression of other correlated genes. Real-time quantitative RT-PCR showing the transcript levels of *CCT3*, *DCUN1D5*, *ENO1*, *MACF1*, or *PPM1G* in SH-SY5Y and SK-N-SH cells stably transfected with empty vector (mock), *ITLN1* **(A)**, sh-Scb, or sh-ITLN1 **(B)**. Additional file 4: Figure S4. Data mining of KLF4 in publicly available tumor database. **(A)** Pearson’s coefficient correlation analysis for the relationship between *KLF4* and *NDRG2* transcript levels in 88 NB tissues derived from R2 microarray analysis and visualization platform. **(B)** Kaplan–Meier survival plots of 88 NB patients with low or high expression of KLF4 derived from R2 microarray analysis and visualization platform. Additional file 5: Figure S5. Restoration of NDRG2 expression in NB cells. Real-time quantitative RT-PCR showing the transcript levels of *ITLN1* and *NDRG2* in NB cells stably transfected with empty vector (mock), *ITLN1*, sh-Scb, or sh-ITLN1, and those co-transfected with sh-NDRG2 **(A)** or NDRG2 **(B)**. **P* < 0.01 vs. mock or sh-Scb. Additional file 6: Figure S6. ITLN1 suppresses the aggressiveness of NB cells through up-regulating NDRG2. The sh-NDRG2 or *NDRG2* was transfected into NB cells stably transfected with empty vector (mock), *ITLN1*, sh-Scb, or sh-ITLN1. The MTT colorimetric assay **(A and D)**, colony formation assay **(B and E)**, and scratch assay **(C and F)** showing the changes in cell viability, growth, and migration. **P* < 0.01 vs. mock or sh-Scb. Additional file 7: Table S1. ITLN1 expression in human NB tissues. Additional file 8: Table S2. Correlation between the expression of ITLN1 and NDRG2. Additional file 9: Figure S7. No epigenetic regulation of ITLN1 in NB cells. Real-time quantitative RT-PCR showing the *ITLN1* transcript levels in NB cells treated with solvent (mock), 5-Aza-CdR (5 μmol/L, **A**), or TSA (200 nmol/L, **B**) for 24 hrs. Additional file 10: Table S3. Primer sets used for qPCR and ChIP. Additional file 11: Table S4. Oligonucleotide sets used for constructs and short hairpin RNAs.
