# Supplementary figures and images for: Intelectin 1 suppresses the growth, invasion and metastasis of neuroblastoma cells through up-regulation of N-myc downstream regulated gene 2
Source: Mol Cancer. 2015 Feb 21;14:47. doi: 10.1186/s12943-015-0320-6 (PMC4359454; doi:10.1186/s12943-015-0320-6)

# Supplementary Figure S1

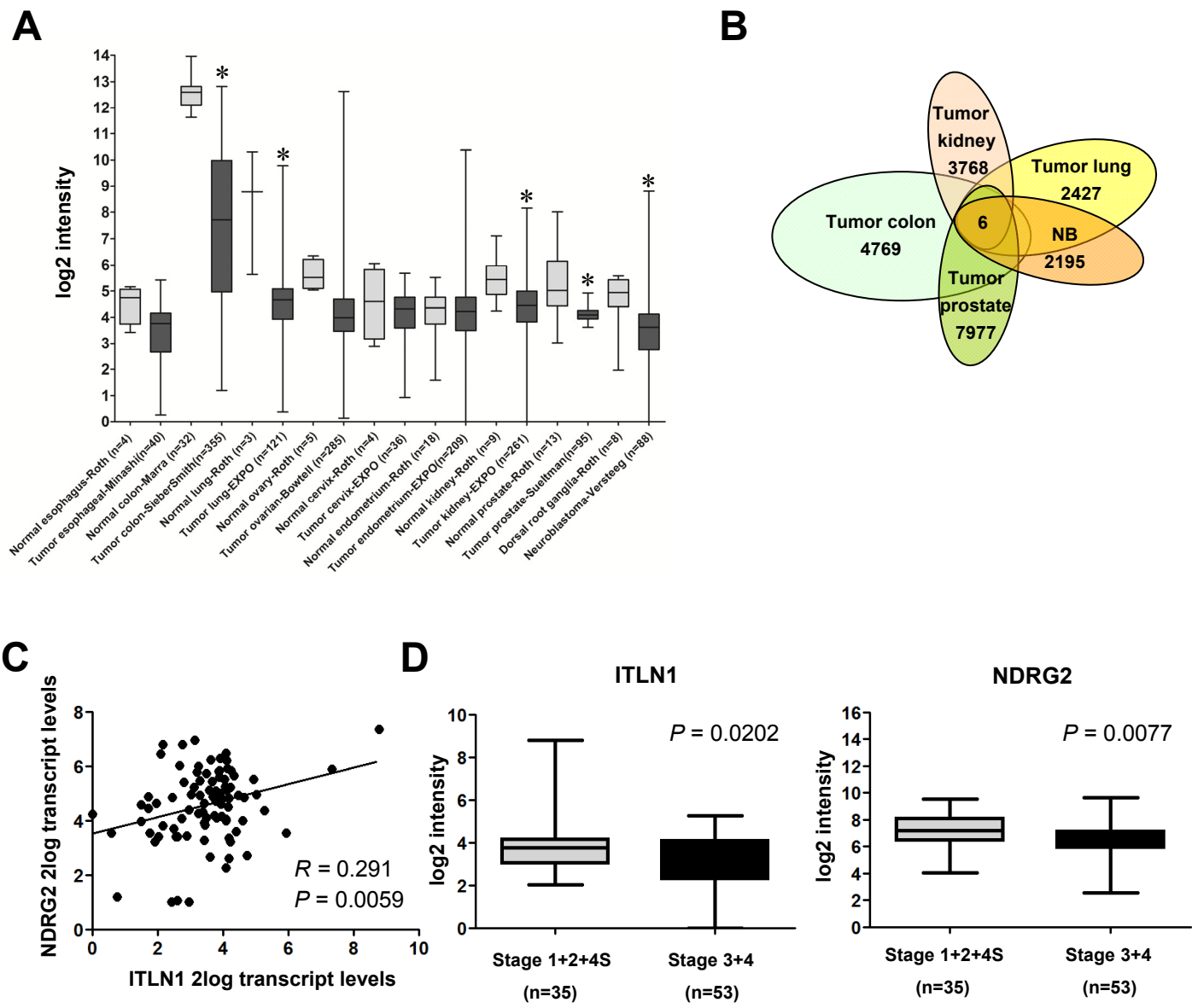

Supplement: Additional file 1: Figure S1. — Data mining in R2: microarray analysis and visualization platform. (A) The ITLN1 transcript levels in different types of normal and tumor tissues. (B) Over-lapping analysis showing six genes significantly correlated with ITLN1 in colon cancer, lung cancer, renal cancer, prostate cancer, and NB, including NDRG2, CCT3, DCUN1D5, ENO1, MACF1, and PPM1G. (C) Pearson’s coefficient correlation analysis for the relationship between ITLN1 and NDRG2 transcript levels in 88 well-defined NB cases. (D) The ITLN1 and NDRG2 transcript levels in NB cases (n = 88) with different INSS stages. *P < 0.05 vs. normal tissues. [file 12943_2015_320_MOESM1_ESM.pdf]

# Supplementary Figure S2

**A**

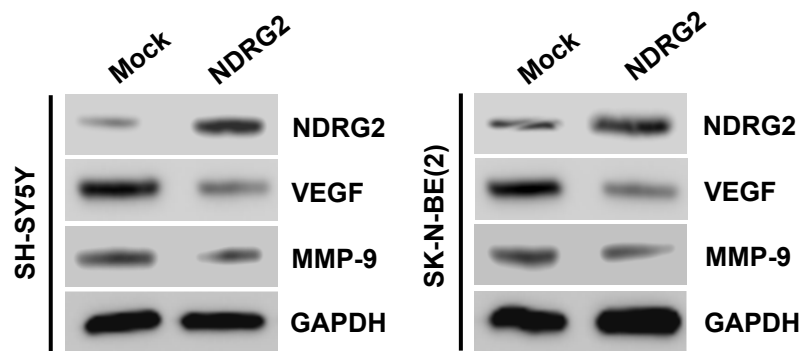

**B**

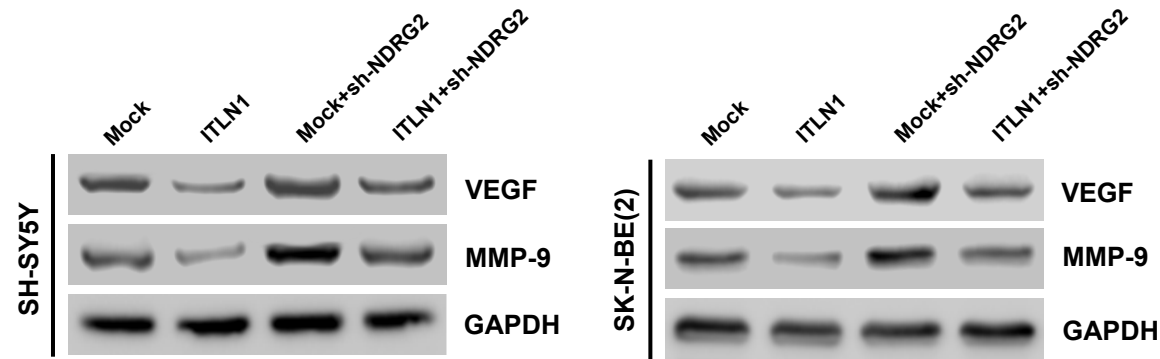

Supplement: Additional file 2: Figure S2. — Direct regulation of VEGF and MMP-9 by NDRG2 in NB cells. (A) Western blot showing the expression of NDRG2, VEGF, and MMP-9 in SH-SY5Y and SK-N-BE(2) cells transfected with empty vector (mock) or NDRG2. (B) Western blot showing the expression of VEGF and MMP-9 in NB cells stably transfected with mock or ITLN1, and those co-transfected with sh-NDRG2. [file 12943_2015_320_MOESM2_ESM.pdf]

# Supplementary Figure S3

A

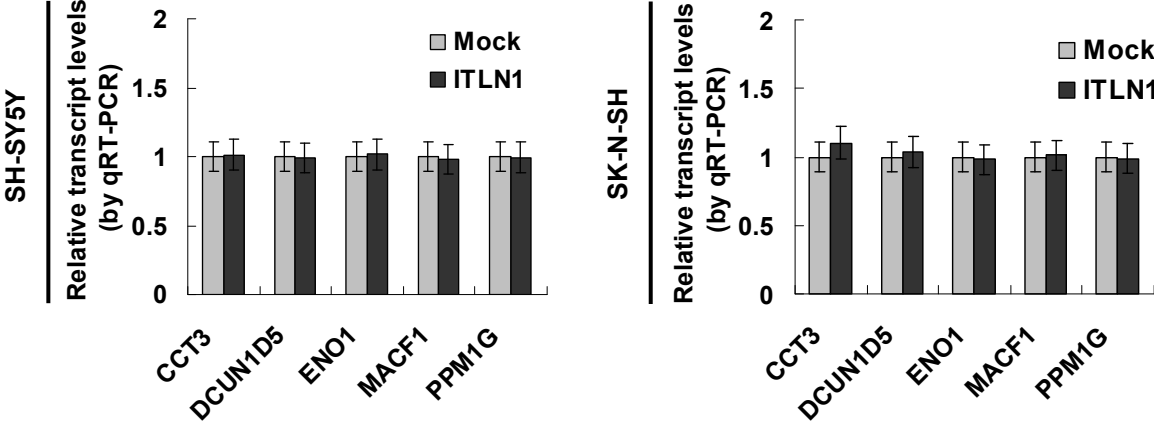

B

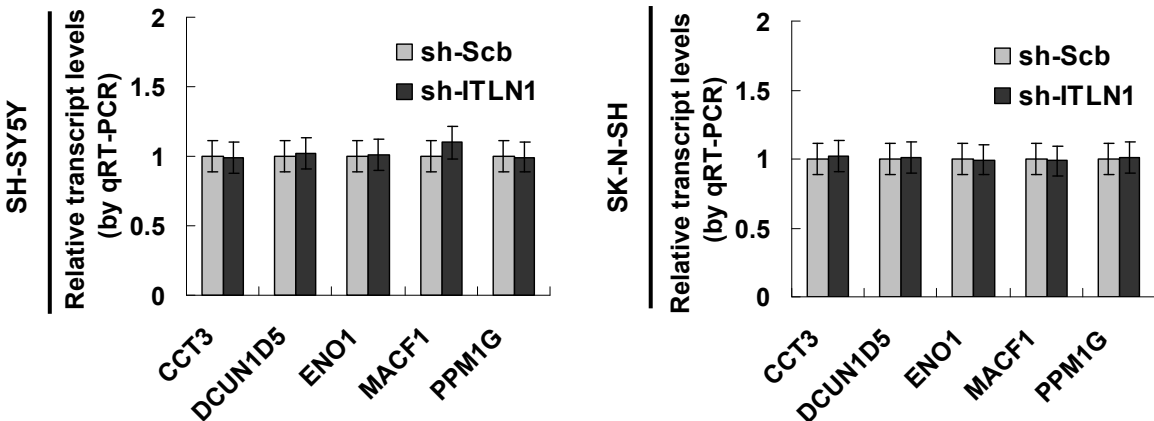

Supplement: Additional file 3: Figure S3. — ITLN1 does not affect the expression of other correlated genes. Real-time quantitative RT-PCR showing the transcript levels of CCT3, DCUN1D5, ENO1, MACF1, or PPM1G in SH-SY5Y and SK-N-SH cells stably transfected with empty vector (mock), ITLN1 (A), sh-Scb, or sh-ITLN1 (B). [file 12943_2015_320_MOESM3_ESM.pdf]

# Supplementary Figure S4

A

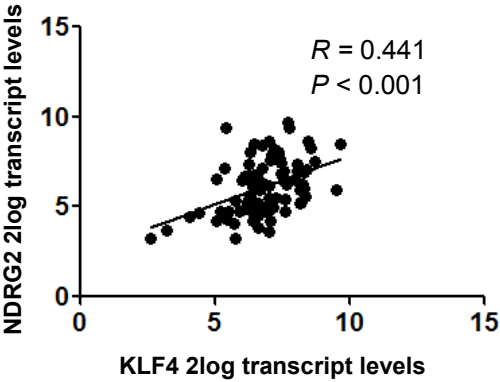

B

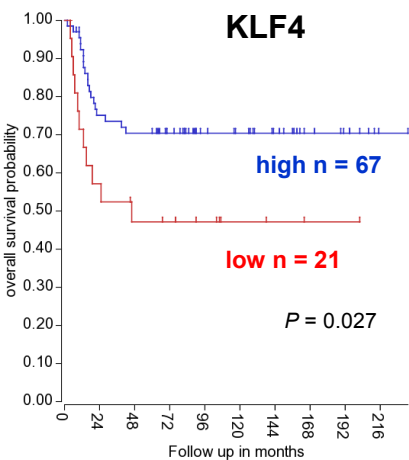

Supplement: Additional file 4: Figure S4. — Data mining of KLF4 in publicly available tumor database. (A) Pearson’s coefficient correlation analysis for the relationship between KLF4 and NDRG2 transcript levels in 88 NB tissues derived from R2 microarray analysis and visualization platform. (B) Kaplan–Meier survival plots of 88 NB patients with low or high expression of KLF4 derived from R2 microarray analysis and visualization platform. [file 12943_2015_320_MOESM4_ESM.pdf]

# Supplementary Figure S5

A

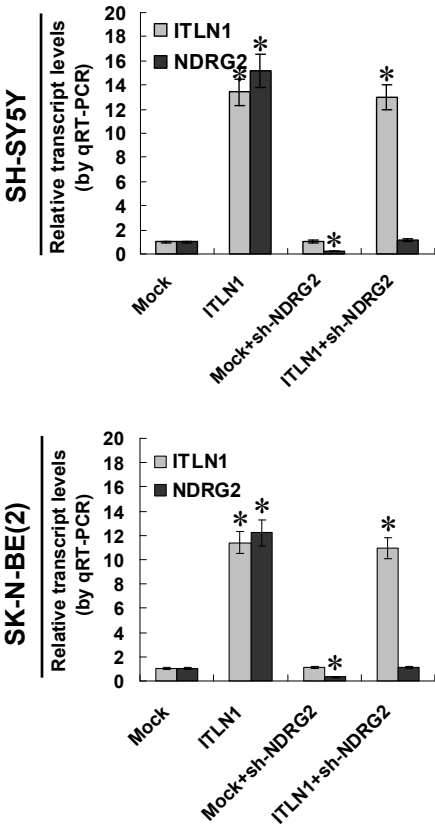

B

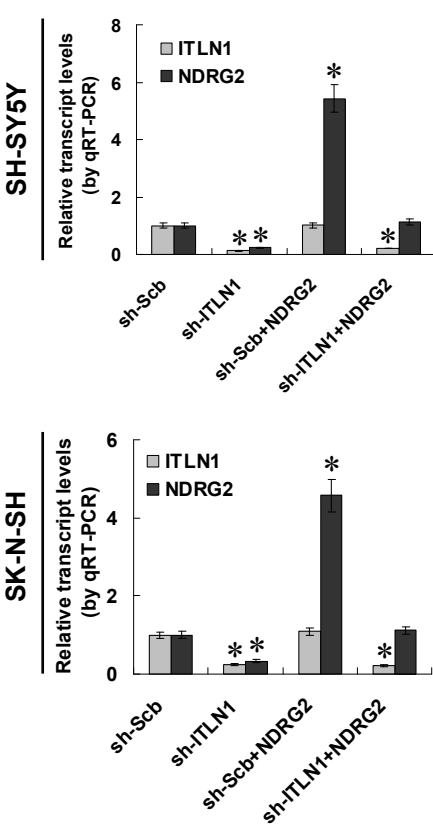

Supplement: Additional file 5: Figure S5. — Restoration of NDRG2 expression in NB cells. Real-time quantitative RT-PCR showing the transcript levels of ITLN1 and NDRG2 in NB cells stably transfected with empty vector (mock), ITLN1, sh-Scb, or sh-ITLN1, and those co-transfected with sh-NDRG2 (A) or NDRG2 (B). *P < 0.01 vs. mock or sh-Scb. [file 12943_2015_320_MOESM5_ESM.pdf]

Supplementary Figure S6

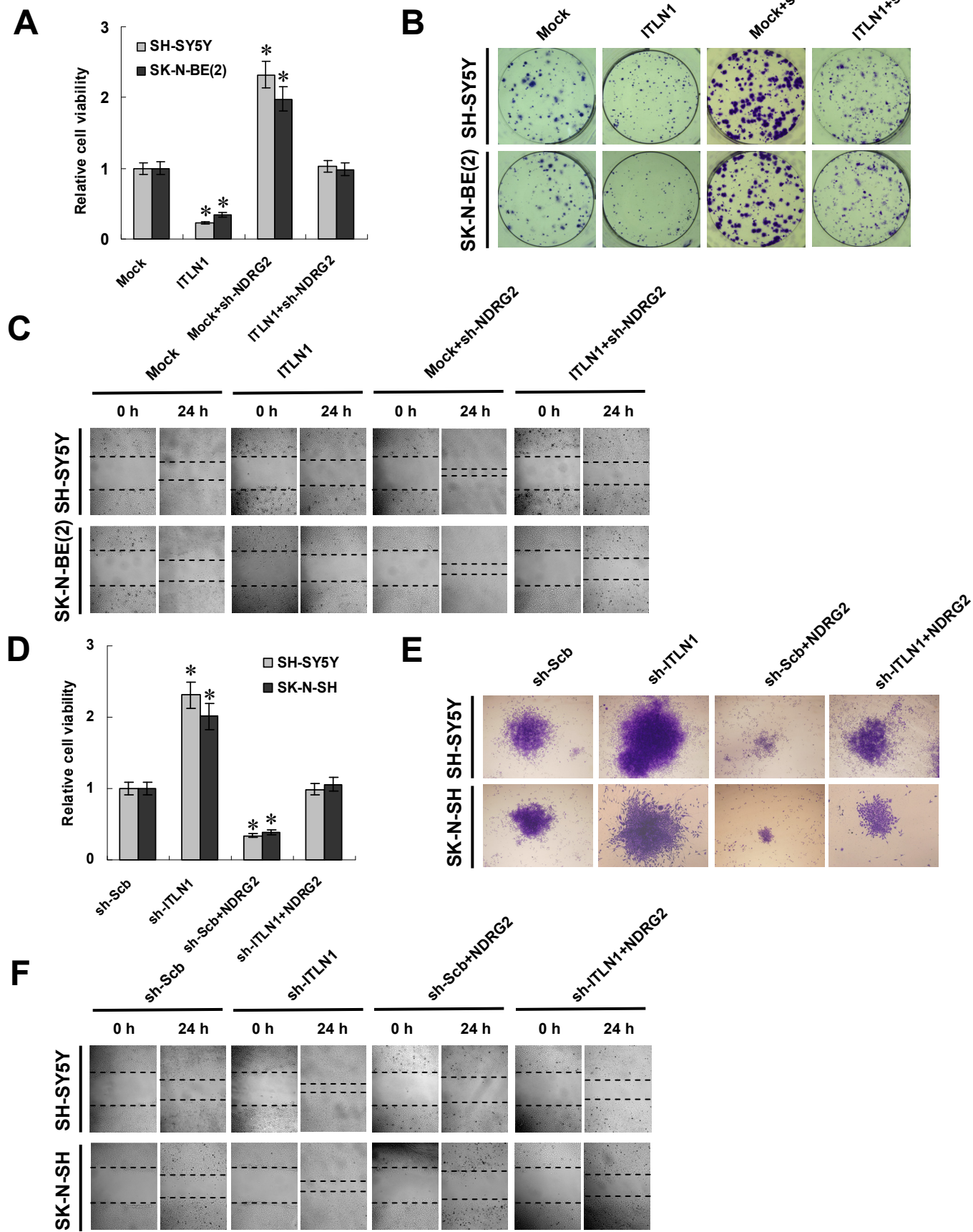

Supplement: Additional file 6: Figure S6. — ITLN1 suppresses the aggressiveness of NB cells through up-regulating NDRG2. The sh-NDRG2 or NDRG2 was transfected into NB cells stably transfected with empty vector (mock), ITLN1, sh-Scb, or sh-ITLN1. The MTT colorimetric assay (A and D), colony formation assay (B and E), and scratch assay (C and F) showing the changes in cell viability, growth, and migration. *P < 0.01 vs. mock or sh-Scb. [file 12943_2015_320_MOESM6_ESM.pdf]

# Supplementary Figure S7

A

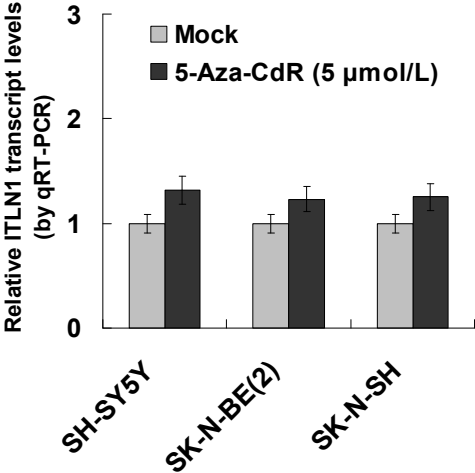

B

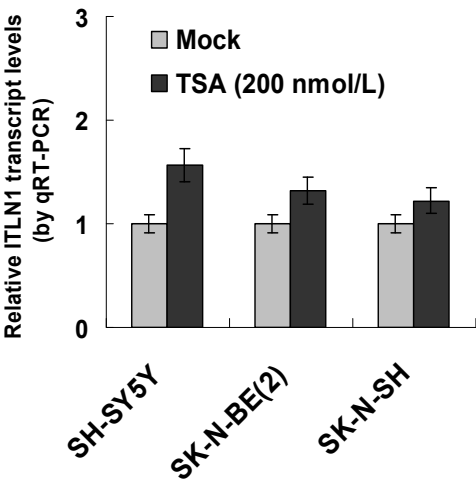

Supplement: Additional file 9: Figure S7. — No epigenetic regulation of ITLN1 in NB cells. Real-time quantitative RT-PCR showing the ITLN1 transcript levels in NB cells treated with solvent (mock), 5-Aza-CdR (5 μmol/L, A), or TSA (200 nmol/L, B) for 24 hrs. [file 12943_2015_320_MOESM9_ESM.pdf]
