# Supplementary material for: Intelectin 1 suppresses the growth, invasion and metastasis of neuroblastoma cells through up-regulation of N-myc downstream regulated gene 2
Source: Mol Cancer. 2015 Feb 21;14:47. doi: 10.1186/s12943-015-0320-6 (PMC4359454; doi:10.1186/s12943-015-0320-6)
Supplement: Additional file 7: Table S1. — ITLN1 expression in human NB tissues. [file 12943_2015_320_MOESM7_ESM.doc]

**Supplementary Table S1 ITLN1 expression in human NB tissues**

| **Group** | **Total number** | **Expression of ITLN1** | | | | **Positive rates** | ***P*-Value** |
| --- | --- | --- | --- | --- | --- | --- | --- |
|  |  | − | + | ++ | +++ | **(%)** |  |
| Age |  |  |  |  |  |  |  |
| < 1 year | 20 | 9 | 4 | 4 | 3 | 55.0 | 0.03 |
| ≥1 year | 22 | 19 | 2 | 1 | 0 | 13.6 |  |
|  |  |  |  |  |  |  |  |
| Differentiation |  |  |  |  |  |  |  |
| Well differentiated | 8 | 0 | 2 | 3 | 3 | 100.0 |  |
| Poorly differentiated | 28 | 23 | 3 | 2 | 0 | 17.9 | <0.001 |
| Undifferentiated | 6 | 5 | 1 | 0 | 0 | 16.7 |  |
|  |  |  |  |  |  |  |  |
| MKI |  |  |  |  |  |  |  |
| < 200 | 17 | 7 | 2 | 5 | 3 | 58.8 | 0.002 |
| > 200 | 25 | 21 | 4 | 0 | 0 | 16.0 |  |
|  |  |  |  |  |  |  |  |
| INSS stages |  |  |  |  |  |  |  |
| Stage 1-2 | 14 | 4 | 3 | 4 | 3 | 71.4 |  |
| Stage 3-4 | 20 | 19 | 1 | 0 | 0 | 5.0 | 0.003 |
| Stage 4S | 8 | 5 | 2 | 1 | 0 | 37.5 |  |

ITLN1, intelectin 1; MKI, mitosis karyorrhexis index; INSS, international neuroblastoma staging system
