# Supplementary material for: Intelectin 1 suppresses the growth, invasion and metastasis of neuroblastoma cells through up-regulation of N-myc downstream regulated gene 2
Source: Mol Cancer. 2015 Feb 21;14:47. doi: 10.1186/s12943-015-0320-6 (PMC4359454; doi:10.1186/s12943-015-0320-6)
Supplement: Additional file 8: Table S2. — Correlation between the expression of ITLN1 and NDRG2. [file 12943_2015_320_MOESM8_ESM.doc]

**Supplementary Table S2 Correlation between the expression of ITLN1 and NDRG2**

|  |  | **ITLN1 expression** | |  |  |  |
| --- | --- | --- | --- | --- | --- | --- |
|  |  | Low | High |  | *R*-value | *P*-value |
| **NDRG2 expression** | |  |  |  |  |  |
|  |  |  |  |  |  |  |
|  | Low | 30 | 1 |  | 0.676 | < 0.001 |
|  |  |  |  |  |  |  |
|  | High | 4 | 7 |  |  |  |

ITLN1, intelectin 1; NDRG2, N-myc downstream regulated gene 2; Pearson’s correlation coefficient

was applied to determine the expression correlation.
