# Supplementary material for: Intelectin 1 suppresses the growth, invasion and metastasis of neuroblastoma cells through up-regulation of N-myc downstream regulated gene 2
Source: Mol Cancer. 2015 Feb 21;14:47. doi: 10.1186/s12943-015-0320-6 (PMC4359454; doi:10.1186/s12943-015-0320-6)
Supplement: Additional file 10: Table S3. — Primer sets used for qPCR and ChIP. [file 12943_2015_320_MOESM10_ESM.doc]

**Supplementary Table S3 Primer sets used for qPCR and ChIP**

| **Primer set** | **Primers** | **Sequence** | **Product size**  **(bp)** | **Application** |
| --- | --- | --- | --- | --- |
| ITLN1 | Forward | 5'-AATGGACCTGTTCTTCGT-3' | 253 | qPCR |
|  | Reverse | 5'-TCTGGGTAGACTGCTTTG-3' |  |  |
| NDRG2 | Forward | 5'-AGCTTGCAGACATGATCCCT-3' | 97 | qPCR |
|  | Reverse | 5'-TATCTCGCCAGGATGTAGGC-3' |  |  |
| VEGF | Forward | 5'-ATGACGAGGGCCTGGAGTGT-3' | 226 | qPCR |
|  | Reverse | 5'-CATTTACACGTCTGCGGATCT-3' |  |  |
| MMP-9 | Forward | 5'-CAGAGATGCGTGGAGAGT-3' | 220 | qPCR |
|  | Reverse | 5'-TCTTCCGAGTAGTTTTGG-3' |  |  |
| KLF4 | Forward | 5'-CAGAGGAGCCCAAGCCAAAG-3' | 220 | qPCR |
|  | Reverse | 5'-TTACGGTAGTGCCTGGTCAGTT-3' |  |  |
| CCT3 | Forward | 5'-ATGACTGGTGTGGAACAA-3' | 112 | qPCR |
|  | Reverse | 5'-GGGAGGTAAGTAGACGGA-3' |  |  |
| DCUN1D5 | Forward | 5'-GAAGAAGAAGAGAAAATCCCCT-3' | 168 | qPCR |
|  | Reverse | 5'-GCATATTCATAAAACCAAGCCA-3' |  |  |
| ENO1 | Forward | 5'-TGCCCTGGTTAGCAAGAA-3' | 190 | qPCR |
|  | Reverse | 5'-AGCCAAGTCAGCGATGTG-3' |  |  |
| MACF1 | Forward | 5'-ACTCATTCACCGATACCGAC-3' | 146 | qPCR |
|  | Reverse | 5'-ATGGCACATCCACATCTTCT-3' |  |  |
| PPM1G | Forward | 5'-AGCAAATCTGGAGGTGGG-3' | 222 | qPCR |
|  | Reverse | 5'-GGCTGAAGAGCAGGAAGG-3' |  |  |
| GAPDH | Forward | 5'-AGAAGGCTGGGGCTCATTTG-3' | 258 | qPCR |
|  | Reverse | 5'-AGGGGCCATCCACAGTCTTC-3' |  |  |
| NDRG2 ChIP | Forward | 5'-TGTTTACCCAGGAGTCAGAGCC-3' | 188 | ChIP |
|  | Reverse | 5'-CGGGAGAAGTTGGACAACAAGG-3‘ |  |  |

ITLN1, intelectin 1; NDRG2, N-myc downstream regulated gene 2; VEGF, vascular endothelial growth factor; MMP-9, matrix metallopeptidase 9; KLF4, Krüppel-like factor 4; CCT3, chaperonin containing TCP1 subunit 3; DCUN1D5, defective in cullin neddylation 1 domain containing 5; ENO1, enolase 1; MACF1, microtubule-actin crosslinking factor 1; PPM1G, protein phosphatase, Mg^2+^/Mn^2+^ dependent, 1G; GAPDH, glyceraldehyde 3-phosphate dehydrogenase.
