# Supplementary material for: Intelectin 1 suppresses the growth, invasion and metastasis of neuroblastoma cells through up-regulation of N-myc downstream regulated gene 2
Source: Mol Cancer. 2015 Feb 21;14:47. doi: 10.1186/s12943-015-0320-6 (PMC4359454; doi:10.1186/s12943-015-0320-6)
Supplement: Additional file 11: Table S4. — Oligonucleotide sets used for constructs and short hairpin RNAs. [file 12943_2015_320_MOESM11_ESM.doc]

**Supplementary Table S4 Oligonucleotide sets used for constructs and short hairpin RNAs**

| **Oligo Set** | **Sequences** |
| --- | --- |
| pcDNA3.1-ITLN1 | 5'-CGCCCAAGCTTATGAACCAACTCAGCTTC-3' (sense); |
|  | 5'-CGCGGATCCTCAACGATAGAATAGAAGCAC-3' (antisense) |
|  |  |
| pcDNA3.1-KLF4 | 5'-CGCCCAAGCTTATGAGGCAGCCACCTGGCGAGT-3' (sense); |
|  | 5'-CGCGGATCCTTAAAAATGCCTCTTCATGTGTAAGGC-3' (antisense) |
|  |  |
| pGL3-NDRG2 | 5'-CCCCCGCCTGCCCGCGATCGATATCTATTTATAG-3' (sense); |
| (ΔKLF4) | 5'-CGATCGCGGGCAGGCGGGGGGTGGGGAGAG-3' (antisense) |
|  |  |
| sh-Scb | 5'-GAGGTAGCGTTTAGTACTTATCTCGAGATAAGTACTAAACGCTACCTC-3' (sense); |
|  | 5'-GAGGTAGCGTTTAGTACTTATCTCGAGATAAGTACTAAACGCTACCTC-3' (antisense) |
|  |  |
| sh-ITLN1 | 5'-GATATGGAACTCATGTTGGTTCTCGAGAACCAACATGAGTTCCATATC-3' (sense); |
|  | 5'-GATATGGAACTCATGTTGGTTCTCGAGAACCAACATGAGTTCCATATC-3' (antisense) |
|  |  |
| sh-NDRG2 | 5'-GAGGACATGCAGGAAATCATTCTCGAGAATGATTTCCTGCATGTCCTC-3' (sense); |
|  | 5'-GAGGACATGCAGGAAATCATTCTCGAGAATGATTTCCTGCATGTCCTC-3' (antisense) |
|  |  |
| sh-KLF4 | 5'-CCGGGCCTTACACATGAAGAGGCATCTCGAGATGCCTCTTCATGTGTAAGGCTTTTTG-3' (sense); |
|  | 5'-AATTCAAAAAGCCTTACACATGAAGAGGCATCTCGAGATGCCTCTTCATGTGTAAGGC-3' (antisense) |

ITLN1, intelectin 1; NDRG2, N-myc downstream regulated gene 2; KLF4, Krüppel-like factor 4; sh-Scb, scramble

short hairpin RNAs
